# Complete Genome Sequence of Acinetobacter nosocomialis GNU001, Isolated from a Plastic-Containing Landfill

**DOI:** 10.1128/mra.01077-22

**Published:** 2023-01-04

**Authors:** Hyeon Jeong Seong, Zhuang Yao, Yu-Sin Jang

**Affiliations:** a Division of Applied Life Science (BK21 Four), Department of Applied Life Chemistry, Institute of Agriculture and Life Science, Gyeongsang National University, Jinju, Republic of Korea; Wellesley College

## Abstract

Due to the hazard of plastic waste exposed to the environment, microorganisms capable of degrading different polymeric pollutants have gained attention. Here, we report the complete genome sequence of Acinetobacter nosocomialis GNU001, which was isolated from a landfill. The genome was composed of a circular chromosome of 3,850,149 bp and a plasmid.

## ANNOUNCEMENT

Plastic waste materials, such as polyethylene, polyethylene terephthalate, polystyrene, and polypropylene, have been buried in landfills; most of the waste is polyethylene (1, 2). Some microorganisms living under landfill conditions are known to degrade such plastic waste materials (3–5). Recently, microorganisms that are capable of degrading plastic pollutants have gained more attention because plastic waste is known to be hazardous to the environment and humans (2, 6). In this study, we analyzed the complete genome sequence of Acinetobacter nosocomialis GNU001, which is capable of degrading polyethylene and was isolated by subculture methods from a polyethylene waste fragment collected at a landfill site.

To extract total DNA, the A. nosocomialis strain GNU001 was cultured overnight at 37°C in Luria-Bertani broth containing 10 g/L peptone, 10 g/L NaCl, and 5 g/L yeast extract. Total DNA was extracted from harvested GNU001 cells using the G-spin genomic DNA extraction kit (iNtRON Biotechnology, Seoul, South Korea), according to the manufacturer’s instructions. The genome of the GNU001 strain was sequenced at Macrogen, Inc. (Seoul, South Korea), using a HiSeq 2500 instrument (Illumina, San Diego, CA, USA) and an RS II system (Pacific Biosciences [PacBio], Menlo Park, CA, USA).

For preparation of the sequencing library, DNA was sheared using adaptive focused acoustics technology (Covaris, Inc., Woburn, MA, USA). Illumina sequencing libraries were prepared from the sheared DNA fragments using the TruSeq Nano DNA kit (Illumina) and sequenced in paired-end format. For long-read sequencing with the PacBio RS II system, libraries were prepared using the SMRTbell preparation kit v1.0 (PacBio). In the software analyses, default values were used for all parameters unless otherwise noted. The Illumina and PacBio raw reads were quality controlled using FastQC v0.11.9 and NanoPlot v1.30.1, respectively. As a result, we obtained 8,235,644 filtered reads, with a total length of 1,243,582,244 bases (99.57% with Q values of ≥20) and a G+C content of 38.45%, from the Illumina high-throughput sequencing. We also obtained 123,014 filtered subreads, with a mean length of 11,503 bp and a total size of 1,415,136,762 bp (*N*_50_, 17,675 bp), from the sequencing using the PacBio RS II system.

Canu v1.7 was used for the draft *de novo* assembly with correction and trimming of the PacBio long reads. Using Canu, circularity was also identified, and the sequence was trimmed if the two ends of the contig overlapped. The assemblies were then corrected using Pilon v1.21 (7) by combining the Illumina reads (2 × 151-bp paired-end reads). The resulting assembly revealed two contigs, i.e., one circular chromosome and one plasmid (Table 1). The chromosome length was 3,850,149 bases, with a G+C content of 38.84%, while the plasmid length was 17,505 bp. Genome annotation was conducted using the Prokaryotic Genome Annotation Pipeline (PGAP) (8). The annotation by PGAP indicated 3,525 and 19 protein-coding genes in the chromosome and the plasmid, respectively. tRNAs and rRNAs were found only in the chromosome, with numbers of 73 and 18, respectively. Further study is in progress to identify the genes responsible for polyethylene degradation, which could be used to develop eco-friendly technology to decompose plastic waste in the environment.

**TABLE 1:** Genome assemblies of A. nosocomialis GNU001, which was isolated from a plastic-containing landfill

Genomic element	Contig no.	Length (bp)	G+C content (%)	Type	No. of CDSs^*a*^	No. of tRNAs	No. of rRNAs
Chromosome	1	3,850,149	38.84	Circular	3,525	73	18
Plasmid	2	17,400	38.97	Circular	19	0	0

a CDS, coding sequence.

### Data availability.

The complete genome sequence has been deposited under the GenBank accession numbers CP107049 (chromosome) and CP107050 (plasmid). The raw sequence reads are available in the Sequence Read Archive (SRA) under SRA accession number SRR21860161.
